# Afterslip Moment Scaling and Variability From a Global Compilation of Estimates

**DOI:** 10.1029/2021JB023897

**Published:** 2022-04-12

**Authors:** R. M. Churchill, M. J. Werner, J. Biggs, Å. Fagereng

**Affiliations:** ^1^ School of Earth Sciences University of Bristol Bristol UK; ^2^ School of Earth and Environmental Sciences Cardiff University Cardiff UK

**Keywords:** afterslip, aseismic, database, scaling, moment

## Abstract

Aseismic afterslip is postseismic fault sliding that may significantly redistribute crustal stresses and drive aftershock sequences. Afterslip is typically modeled through geodetic observations of surface deformation on a case‐by‐case basis, thus questions of how and why the afterslip moment varies between earthquakes remain largely unaddressed. We compile 148 afterslip studies following 53 *M*_*w*_6.0–9.1 earthquakes, and formally analyze a subset of 88 well‐constrained kinematic models. Afterslip and coseismic moments scale near‐linearly, with a median Spearman's rank correlation coefficient (CC) of 0.91 after bootstrapping (95% range: 0.89–0.93). We infer that afterslip area and average slip scale with coseismic moment as $M_{o}^{2/3}$ and $M_{o}^{1/3}$, respectively. The ratio of afterslip to coseismic moment (*M*_*rel*_) varies from <1% to >300% (interquartile range: 9%–32%). *M*_*rel*_ weakly correlates with *M*_*o*_ (CC: −0.21, attributed to a publication bias), rupture aspect ratio (CC: −0.31), and fault slip rate (CC: 0.26, treated as a proxy for fault maturity), indicating that these factors affect afterslip. *M*_*rel*_ does not correlate with mainshock dip, rake, or depth. Given the power‐law decay of afterslip, we expected studies that started earlier and spanned longer timescales to capture more afterslip, but *M*_*rel*_ does not correlate with observation start time or duration. Because *M*_*rel*_ estimates for a single earthquake can vary by an order of magnitude, we propose that modeling uncertainty currently presents a challenge for systematic afterslip analysis. Standardizing modeling practices may improve model comparability, and eventually allow for predictive afterslip models that account for mainshock and fault zone factors to be incorporated into aftershock hazard models.

## Key Points

- We compile 148 afterslip studies after 53 earthquakes to probe the scaling and variability of aseismic afterslip moment
- Afterslip moment scales near‐linearly with the coseismic moment but varies from <1% to >300% of coseismic moment between earthquakes
- Different modeling approaches appear to be the dominant cause of variability, but fault slip rate and rupture shape may exert some control

## 1 Introduction

Following an earthquake, various postseismic mechanisms act to relax and redistribute stress concentrations in the crust and upper mantle (Freed, 2005). In addition to seismic aftershocks, postseismic mechanisms include aseismic afterslip (Bürgmann et al., 1997; Marone et al., 1991; Shen et al., 1994), pore fluid flow or poroelastic rebound (Peltzer et al., 1996, 1998; Piombo et al., 2005) and deeper viscoelastic relaxation or viscous flow (Deng et al., 1998; Freed & Lin, 2001; F. F. Pollitz et al., 2001). These processes may generate geodetically observable surface deformation (e.g., with GNSS, InSAR), which can be modeled to provide insight into fault zones, crustal structure, and the earthquake cycle (e.g., Ingleby & Wright, 2017; Massonnet et al., 1994).

Aseismic afterslip may provide particularly valuable insight into fault zone rheology and earthquake cycle processes (Avouac, 2015; Bürgmann, 2018). Afterslip is transient, fault scale, aseismic shear that occurs on and close to the fault planes of the parent earthquake, as postseismic readjustment (Avouac, 2015; Harris, 2017), distinct from generally deeper and more distributed viscoelastic relaxation (K. Wang et al., 2012). Aseismic afterslip is also distinct from seismic aftershocks and is specifically a response to coseismic stress concentrations, thus is also a distinct mechanism from triggered slow slip, which is driven by stresses that have built up over longer timescales (Bürgmann, 2018). Afterslip is globally widespread and relatively easy to detect as the associated surface deformation is initially greater and more near‐field than that caused by viscoelastic relaxation (Diao et al., 2014; Reilinger et al., 2000). There is also mounting evidence that afterslip may drive aftershock sequences (Bürgmann et al., 2002; Hsu et al., 2006; Huang et al., 2017; Peng & Zhao, 2009; Perfettini & Avouac, 2004), therefore it is highly desirable to better understand the phenomenon.

First order behaviors of afterslip, such as the scaling of afterslip moment with coseismic moment, are still poorly understood. Some existing studies have considered afterslip following multiple earthquakes but have been limited in scope. For example, Lange et al. (2014) compared afterslip models for three large subduction thrust events, whilst Hawthorne et al. (2016) and Alwahedi and Hawthorne (2019) analyzed afterslip following *M*_*w*_ < 5 Californian earthquakes. Wimpenny et al. (2017) and Alwahedi and Hawthorne (2019) compiled afterslip moment estimates for approximately 30 global earthquakes and showed that relative afterslip moment (*M*_*rel*_), defined as:

(1) $$M_{rel}=\frac{M_{o}^{aft}}{M_{o}},$$

where $M_{o}^{aft}$ is the afterslip moment and *M*_*o*_ is the coseismic moment, can vary by up to two orders of magnitude between different earthquakes. It is not clear what drives this, for example, can we explain why the similar‐magnitude El Mayor Cucapah and Landers earthquakes generated *M*_*rel*_ of 74% and 2%, respectively (Fialko, 2004; Rollins et al., 2015)? A global synthesis of studies is needed to better establish the average and outlying behaviors of afterslip and provide observational constraints for physical models.

We compile 148 aseismic afterslip studies that follow 53 *M*_*w*_6.0–9.1 earthquakes from 1979 to 2018. Using a refined subset of 88 better‐constrained kinematic afterslip models (after 46 earthquakes), we investigate whether afterslip and coseismic moment scale in a discernible way. We explore whether observed variability in *M*_*rel*_ depends on characteristics of the mainshock (moment, rake, dip, depth, rupture aspect), measures of local deformation rate (fault slip rate, local strain rate, plate velocity), and data availability (the start date and duration of data collection). We discuss additional factors that are difficult to quantify and test statistically, including fault zone composition, earthquake history, and the influence of data availability and modeling methodology. We also investigate whether the occurrence of updip or downdip afterslip may be influenced by vertical rupture directivity, measured by a one‐dimensional estimate. Determining what controls *M*_*rel*_ variation may offer new empirical constraints on afterslip, which could lead to improved predictive models of stress transfer for aftershock modeling and forecasting (Cattania et al., 2015; Mancini et al., 2020).

In Section 2, we outline the observations, kinematics, and a mechanical interpretation of afterslip and formulate hypotheses regarding the potential factors that *M*_*rel*_ might depend on, to later test. In Section 3, we explain our compilation and statistical methods and describe our database, which includes study and model metadata, and information on the mainshock and fault zone setting. This database is available online (doi:10.5281/zenodo.6414330). We present our analysis of the database in Section 4 and discuss our findings in Section 5.

## 2 Background

### 2.1 Observations and Mechanical Interpretation

**Figure 1:**
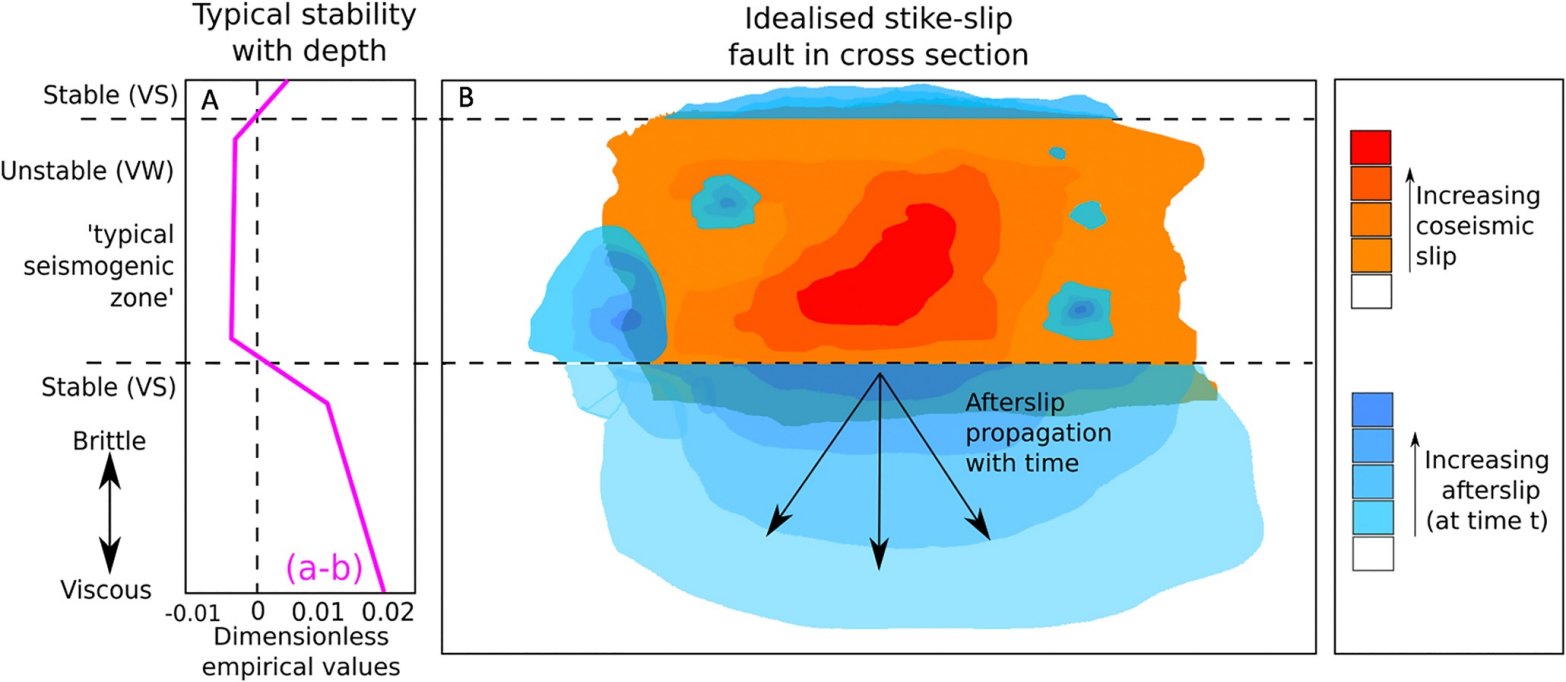
(a) A simplified frictional slip stability (a)–(b) profile with depth (modified from the study by Avouac [2015]; Bürgmann [2018]; Hillers et al. [2006]; Perfettini & Avouac [2007]), and (b), a schematic fault‐parallel cross‐section of idealized coseismic rupture and aseismic afterslip on a well‐coupled strike slip fault. The approximate seismic‐aseismic transition and brittle‐viscous/ductile transitions are shown, but conditional stability is not shown for simplicity.

The kinematics of afterslip can be well approximated by combining a constitutive framework for shear strength (*τ*) at an interface with elastic theory (Rubin, 2008). Rate and state dependent friction describes *τ* and the conditions under which materials strengthen or weaken with an imposed velocity step (Dieterich, 1979, 1987; Ruina, 1983). The Dieterich‐Ruina formulation gives *τ* as:

(2) $$\tau =\sigma \left(\mu_{o}+a\;\ln\;\frac{V}{V_{o}}+b\;\ln\;\frac{V_{o}\theta }{D_{c}}\right),$$

where *σ* is the effective normal stress and *μ*_*o*_ is the friction coefficient when slip velocity (*V*) equals the reference velocity (*V*_*o*_). The direct effect term (*a* ln(*V*/*V*_*o*_)) describes an initial frictional strength increase and the evolution term (*b* ln(*V*_*o*_ *θ*/*D*_*c*_)) describes a frictional strength reduction over slip distance and time, where *D*_*c*_ is the critical slip distance and *θ* is the state variable. *a* and *b* are empirical, dimensionless quantities weighing these terms. *a* − *b* expresses the velocity dependence of a material under given environmental conditions, including stress, temperature, slip velocity, and effects of fluids (Blanpied et al., 1991; Marone, 1998). *a* − *b* is depth‐dependent (Blanpied et al., 1991), with distinct and strongly frictionally stable regions typically updip and downdip of the seismogenic zone, as shown in Figure 1a (e.g., Hillers et al., 2006; Imber et al., 2001). Rate and state dependent friction does not imply a microscale mechanism (Van den Ende et al., 2018), but one key interpretation of afterslip is a brittle creep (e.g., Marone et al., 1991; Perfettini & Avouac, 2004).

In brittle creep interpretations, afterslip occurs principally in frictionally stable fault regions, where (*a* − *b*) > 0 (Marone et al., 1991; Perfettini & Avouac, 2004). Here, seismic nucleation is prohibited and small increments of immediately‐arrested brittle failure (Perfettini & Avouac, 2004) erode away at the stress concentrations left by an earthquake to produce aseismic, macroscale fault slip over time (Bürgmann, 2018; Harris, 1998). As the direct effect term dominates, afterslip can be approximated by a steady‐state process (Marone et al., 1991; Scholz, 1998):

(3) $$\tau =\sigma \left(\mu_{o}+\left(a-b\right)\;\ln\;\frac{V}{V_{o}}\right).$$

Figure 1b shows an idealized schematic of coseismic rupture and afterslip on a well coupled strike slip fault. Here, coseismic rupture is mostly confined to the well‐defined frictionally unstable seismogenic zone but may propagate into adjacent stable regions through dynamic weakening (Noda & Lapusta, 2013; Shaw & Wesnousky, 2008). Afterslip then migrates away from the rupture edges within the frictionally stable regions (Bie et al., 2014; Peng & Zhao, 2009), but some occurs at traditionally seismogenic depths due to rheological heterogeneity or conditional stability. Afterslip has often been observed at traditionally seismogenic depths (e.g., Langbein et al., 2006; Reilinger et al., 2000; Riva et al., 2007), and thus is clearly not limited to distinct and strongly velocity‐strengthening regions (Bürgmann et al., 2002; Helmstetter & Shaw, 2009). In the case of a poorly‐coupled fault, such as the creeping section of the San Andreas (Bürgmann, 2018; Jolivet et al., 2015), isolated velocity‐weakening rupture patches may exist within an overall more velocity strengthening fault. In another case, where fault material is only weakly velocity‐weakening or conditionally stable, where (*a* − *b*) ≈ 0 or <0, aseismic slip may occur if the slip velocities (Scholz, 1998) or the nucleation length scales (related to *D*_*c*_; Boatwright & Cocco, 1996; Bürgmann, 2018; Rubin & Ampuero, 2005) required for seismic slip are not reached. In this case, the steady‐state approximation does not hold, and triggered slow slip events may also be common (Bürgmann, 2018; Rolandone et al., 2018; Taira et al., 2014; Wallace et al., 2018).

### 2.2 Afterslip and the Mainshock and Fault Setting

The nature of a scaling relationship between the afterslip moment and the coseismic moment is not well constrained. Establishing what the expected or average afterslip moment for a given earthquake should be would allow for more informed investigations into behavioral variation. Moment (*M*_*o*_) is given by the product of shear modulus (*G*), average slip $\left(\bar{D}\right)$ and slip area (*A*):

(4) $$M_{o}=GA\bar{D}.$$

Coseismic ruptures are commonly assumed to scale self‐similarly, whereby *M*_*o*_, $\bar{D}$ and *A* grow in a consistent and scale‐invariant way (e.g., Leonard, 2014; Wells & Coppersmith, 1994). Given this, and assuming that coseismic static stress change drives afterslip, we posit a monotonic relationship between afterslip and coseismic moments. Basic elastic theory predicts that the magnitude of stress change around a rupture and the area on the fault plane exposed to a given stress change increase with the coseismic moment (Segall, 2010). Therefore, assuming that shear modulus (*G*) remains approximately constant across seismogenic and afterslip zones, the average slip, area, and overall moment of afterslip should increase with the coseismic moment.

If *M*_*rel*_ is observed to vary, we can investigate factors that might drive this through testing the following hypotheses. If *a* − *b* principally controls afterslip occurrence throughout the fault zone (e.g., Marone et al., 1991; Perfettini & Avouac, 2004) and is largely controlled by temperature and depth (e.g., Blanpied et al., 1991; Hillers et al., 2006; Imber et al., 2001), we hypothesize that low dip angle faults may permit more afterslip, by providing a greater area of unruptured fault in purely and conditionally frictionally stable regions. We also could expect a relationship with a rake, as this typically correlates with dip (Anderson, 1905). Again, assuming that *a* − *b* is depth controlled, we hypothesize that shallower earthquakes may permit more downdip, and therefore overall, afterslip. However, this is complicated by the fact that updip afterslip can occur and that deep ruptures might be required to activate deeper frictionally stable regions in the first place. Finally, Hawthorne et al. (2016) alluded to a potential link between rupture elongation and *M*_*rel*_, thus we investigate the influence of coseismic moment and the aspect ratio (length to downdip width) of coseismic rupture on *M*_*rel*_. We, therefore, investigate relationships between *M*_*rel*_ and mainshock moment, fault dip, rake, depth, and rupture aspect.

Rheology may vary across different fault zones. We hypothesize that mature faults might promote more afterslip as they are suggested to contain higher proportions of velocity‐strengthening materials like gouges and smoothed asperities (Choy & Kirby, 2004; Collettini et al., 2019; Ikari et al., 2011; Imber et al., 2008). Fluids or specific materials that might promote aseismic slip might also be present, such as the talc‐bearing serpentinites in the creeping section of the San Andreas fault (Moore & Rymer, 2007) or well‐connected phyllosilicate gouges (Niemeijer, 2018). We use measures of local deformation rate: fault slip rate (i.e., the long term rate at which a fault slips), local strain rate (i.e., how localized deformation is, a combination of fault zone width and slip rate), and plate velocity, as proxies for fault maturity and potential for abundant (*a* − *b*) >0 material, as there is evidence that factors such as fault slip rate are linked to maturity (e.g., Goldberg et al., 2020; Manighetti et al., 2007). We, therefore, investigate relationships between *M*_*rel*_ and fault slip rate, local strain rate, and plate velocity.

Certain additional factors that may influence *M*_*rel*_ cannot be easily statistically tested. For example, the size and shape of different coseismic ruptures can vary at the same fault patch throughout multiple earthquake cycles (e.g., Bakun et al., 2005; Jiang & Lapusta, 2016; Shaw & Wesnousky, 2008), which may influence subsequent postseismic behaviors, as indicated in some earthquake cycle simulations (e.g., Barbot et al., 2012). This implies that any single observed earthquake and afterslip episode may not reflect the average behavior of events at that fault. Additionally, the variable presence and role of conditionally stable regions across different faults may also drive variations in *M*_*rel*_. This includes whether these regions are locked or creep interseismically, whether they can rupture coseismically, or whether they can fail in either or both spontaneous or triggered slow slip events (e.g., Scholz, 1998; Noda & Lapusta, 2013; M. Wei et al., 2013; Bürgmann, 2018). Finally, the interseismic coupling may be linked with *M*_*rel*_ through factors such as fault maturity, rheology, fluid pressure, and structural heterogeneity (Chaussard et al., 2015; Harris, 2017; Kaneko et al., 2010). A lack of reliable interseismic coupling estimate at many host faults makes this difficult to evaluate but is desirable for the future. These factors will be discussed alongside the implications of our results in Section 5.2.

### 2.3 Methods and Limitations of Observation and Modeling

Our understanding of afterslip derives principally from geodetic observations of its surface deformation (Bürgmann, 2018). The broad types of data used to analyze afterslip are ground‐based surveys (e.g., creep‐ and strainmeters, etc.), GNSS (including GPS), InSAR, and satellite gravimetry. As these observation methods have absolute detection thresholds, we expect a bias in the literature toward readily detectable afterslip episodes. This could manifest as an apparent dependence of *M*_*rel*_ on mainshock magnitude, as low *M*_*rel*_ following large earthquakes may be detectable, whereas low *M*_*rel*_ following smaller earthquakes may not. Additionally, the deformation signals of different postseismic mechanisms may be overlain and concurrent (Barbot & Fialko, 2010), making it difficult to distinguish their individual contributions (e.g., Biggs et al., 2009; Ryder et al., 2007). Separating the contributions of afterslip and viscoelastic relaxation becomes particularly difficult above mainshock magnitudes *M*_*w*_6.5‐7, and the two processes can trade off strongly in models (e.g., Jacobs et al., 2002; Sun & Wang, 2015; Luo & Wang, 2021; M. Wang et al., 2021).

Afterslip moment estimates will be sensitive to the temporal window of observation. The steady‐state approximation predicts afterslip velocity *V* at time *t* as:

(5) $$V(t)=\frac{V_{0}}{1+\frac{t}{C}},$$

where *V*_0_ is the initial velocity and *C* is a constant of decay. This approximation is well supported by observations, where the afterslip signal has a high onset amplitude (e.g., S. Wei et al., 2015; Tsang et al., 2019) and decays approximately with the inverse of time (e.g., Azúa et al., 2002; Ingleby & Wright, 2017; Marone, 1998; Perfettini & Avouac, 2004; Wennerberg & Sharp, 1997), and implies that the earliness and duration of study are crucial for capturing a representative afterslip signal.

Afterslip studies fall into three broad categories, each of which has different outputs and implications for this analysis. First, geodetic analyses are studies that typically fit decay equations to surface displacements (e.g., Savage & Svarc, 2009) or estimate the first‐order spatial extent of afterslip (e.g., Ergintav et al., 2007), but do not produce a spatial distribution model of afterslip. Their conclusions regarding the spatial distribution of afterslip are generally qualitative and do not include a moment estimate. Kinematic slip modeling refers to studies that fit a spatial slip model to geodetic observations through dislocation theory (Okada, 1992; Segall, 2010). This may involve iterative forward modeling (e.g., Reilinger & Larsen, 1986) or explicit numerical inversion (e.g., L. Wang et al., 2009; Menke, 2018). Finally, dynamic slip modeling refers to studies that use a nonlinear inversion to constrain frictional parameters within frameworks such as the steady‐state approximation. These can then produce a model of evolving afterslip from an initial postseismic stress field, which also satisfies geodetic observations (e.g., Johnson et al., 2009; Perfettini & Avouac, 2007). The inversion process is associated with considerable uncertainty arising from the validity of assumptions, inherent non‐uniqueness, and regularization (Scales & Tenorio, 2001), discussed further in Section 5.3.

## 3 Data Compilation and Methods

### 3.1 Compilation From the Literature

We compile afterslip studies that follow *M*_*w*_6.0 or greater earthquakes from 1979 onward, published until 2018 (inclusive). We omit earthquakes before this due to poor data quality, notably excluding: the 1959 Hebgen Lake (Nishimura & Thatcher, 2003), the 1978 Tabas E Golshan (Copley, 2014), and the 1940 Imperial Valley (Reilinger, 1984) earthquakes. The inclusion of a study in our compilation is irrespective of whether additional postseismic mechanisms are considered, but we note when viscoelastic relaxation and pore fluid effects are considered or modeled.

We systematically extract information about the afterslip model(s) from each study. We identify each study's preferred afterslip model, proposed by the authors as the best compromise of physical sense and data fit and record the proposed moment, any bounds on this (from error analysis or viable alternative models), and the depth extent of ‘most’ afterslip. The latter is approximate and often derived from qualitative discussions or inferred from figures, as digitized afterslip models are scarcely provided. We omit one‐moment estimate from a study by Paul et al. (2007) because it considers only a proportion of the total spatial extent of afterslip and would not be comparable. If multiple viable models are proposed without a strong preference, we average the proposed moments. For magnitude‐moment conversions we use Hanks and Kanamori (1979), where *M*_*o*_ is in N m (Nm):

(6) $$M_{w}=\left(\log_{10}\;M_{o}-9.05\right)/1.5.$$

We assume that a significant deformation signal related to aftershocks has been removed (e.g., Hoffmann et al., 2018; Howell et al., 2017) or is negligible (e.g., Barnhart et al., 2016; Béjar‐Pizarro et al., 2010). However, seismic afterslip and aftershocks are not treated as separate mechanisms in some studies. To consistently consider only aseismic afterslip, we reduce the moment estimates of Donnellan et al. (2002), Gahalaut et al. (2008), and Shrivastava et al. (2016) by 13%, 47%, and 10%, respectively, which they explicitly gave as the seismic proportions of their afterslip moment estimates.

We record data and modeling information for each study. This includes the data type(s) used, the start and end time of observation (converted to an approximate number of days since mainshock), the broad modeling type, and many individual modeling choices, where possible (see supplementary materials or database for detail). We assume a start time of 1 day when one is not explicitly given, as these are generally continuous GPS studies, and/or we assume that longer delays between the parent earthquake and data collection would be mentioned explicitly. Some studies also account for early missing afterslip by extrapolation (e.g., D’Agostino et al., 2012; Perfettini et al., 2010) or by estimating how much afterslip is contained within the coseismic model (e.g., Hutton et al., 2001), and we use these estimates.

### 3.2 Compilation of Mainshock Data

We compile mainshock information from global earthquake catalogs. For each earthquake, we record the moment, magnitude, longitude, latitude, depth, dip, and rake from the preferred W‐phase moment tensor (*M*_*ww*_) solution of the USGS ComCat database (U.S. Geological Survey, 2017) and from the Global Centroid Moment Tensor (GCMT) catalog (Dziewonski et al., 1981; Ekström et al., 2012). In this study, we do not need to distinguish between left and right‐lateral strike slip, thus we convert the circular rake values to semi‐circular values, with normal and thrust faulting as endmembers and strike slip in between. To deduce the correct fault plane from the two nodal planes of each focal mechanism, we use figures and dip and strike values given in the compiled literature. We obtain a hypocentral depth and an approximate coseismic slip depth extent, bounded by at least 1 cm of slip, from coseismic slip models in the Earthquake Source Model database: SRCMOD (Mai & Thingbaijam, 2014). We use slip models by Hayes (2017) where possible, but otherwise choose a simple, preferably single fault plane model to be as systematic as possible.

In most cases, we use the USGS ComCat preferred solution's seismic moment as the ‘driving’ moment of afterslip. However, for the following cases where the mainshock is ambiguous to define (i.e., mainshock sequences), we use a summed driving moment: (a) the six *M*_*w*_5.2–6.3 1994 Sefidabeh earthquakes, (b) the two *M*_*w*_6.5 2000 South Iceland earthquakes, (c) the *M*_*w*_7.1 2005 Miyagi mainshock and its *M*_*w*_6.6 aftershock, after Miura et al. (2006), (d) the entire 2009 Karonga swarm, as given by Hamiel et al. (2012), (e) the *M*_*w*_8.1 and *M*_*w*_8.3 2006/7 Kuril islands earthquakes, and (f) the *M*_*w*_5.7 and *M*_*w*_6.0 1997 Umbria‐Marche earthquakes. We divide each afterslip moment estimate by the driving moment to obtain *M*_*rel*_.

### 3.3 Compilation of Tectonic Data

We obtain tectonic and fault setting information for each earthquake from external, global data sets. We identify the major fault closest to each mainshock hypocenter in the Global Earthquake Model Foundation (GEM) global active faults database (Styron & Pagani, 2020) and extract the net fault slip rate (i.e., long term average value in the direction of maximum displacement) for each earthquake from the GEM data‐base. We calculate the second invariant of the strain rate tensor closest to the mainshock hypocenter from Kreemer et al. (2014) for continental events. For subduction events, the projection of the hypocenter to the surface is generally far from the fault trace which caused issues in selecting a representative strain rate value systematically. Instead, we obtain a value for plate velocity from the GEM Strain Rate Model: GSRM 2.1 (Kreemer et al., 2014; UNAVCO, 2021) at the hypocentral location of each earthquake, as this is more meaningful.

### 3.4 Statistical Tests

We investigate variations in absolute and relative afterslip moment and test for correlations between relative afterslip moment and various factors. We compile 95 moment estimates from individual studies, but formally analyze a slightly reduced data set of 88 well‐constrained kinematic slip model estimates that follow 46 earthquakes. This small reduction ensures standardization and comparability between the models we analyze.

As there are multiple moment estimates for some events, we bootstrap to fairly sample data and robustly test correlations. For each test, we create 2000 subsets of data, each with one randomly sampled estimate for every earthquake (*n* = 46, the number of earthquakes and data points of each subset). We calculate Spearman's rank correlation coefficient between each subset and the characteristic we are testing and present the median value and 95% range of the distribution. We use Spearman's rank to test for monotonic relationships (Dodge, 2008), as testing specifically for linearity (i.e., Pearson's) may miss complex, nonlinear relationships and could be disproportionately affected by outliers in our data. As the bootstrapped distributions are not necessarily Gaussian, we use the median and 95% range rather than the mean and standard deviation, which could be less representative and more sensitive to outlying values. As our correlation coefficients are based on rank rather than absolute value, we cannot provide a data‐fit or measure of an error on individual coefficients, thus our statistical measures do less well at reflecting the additional uncertainty in individual moment estimates, but we discuss these uncertainties further in Section 4.3. We interpret a result as statistically interesting if the entire 95% range does not cross the zero coefficient line.

We use reduced data sets with specific criteria to further probe the relationships between $M_{o}^{aft}$ and *M*_*o*_, and *M*_*rel*_ and *M*_*o*_. The following reduced data sets contain one estimate per earthquake and do not need bootstrapping: (a) the model with the longest duration for each earthquake (*n* = 45), (b) the largest afterslip moment estimate for each earthquake (*n* = 45), and (c) the longest duration model that also starts within 1 day of the earthquake (*n* = 32). Data set 3 is further refined as (e) removing the two outlying *M*_*rel*_ endmembers (*n* = 30), (f) including only subduction events (*n* = 17), and (g) including only earthquakes *M*_*w*_7.0 or greater. The Sefidabeh study by Copley (2014) is an extreme outlier in terms of the start time (more than 2 years), that we omit in all of these reduced data sets.

## 4 Results

### 4.1 The Database

**Figure 2:**
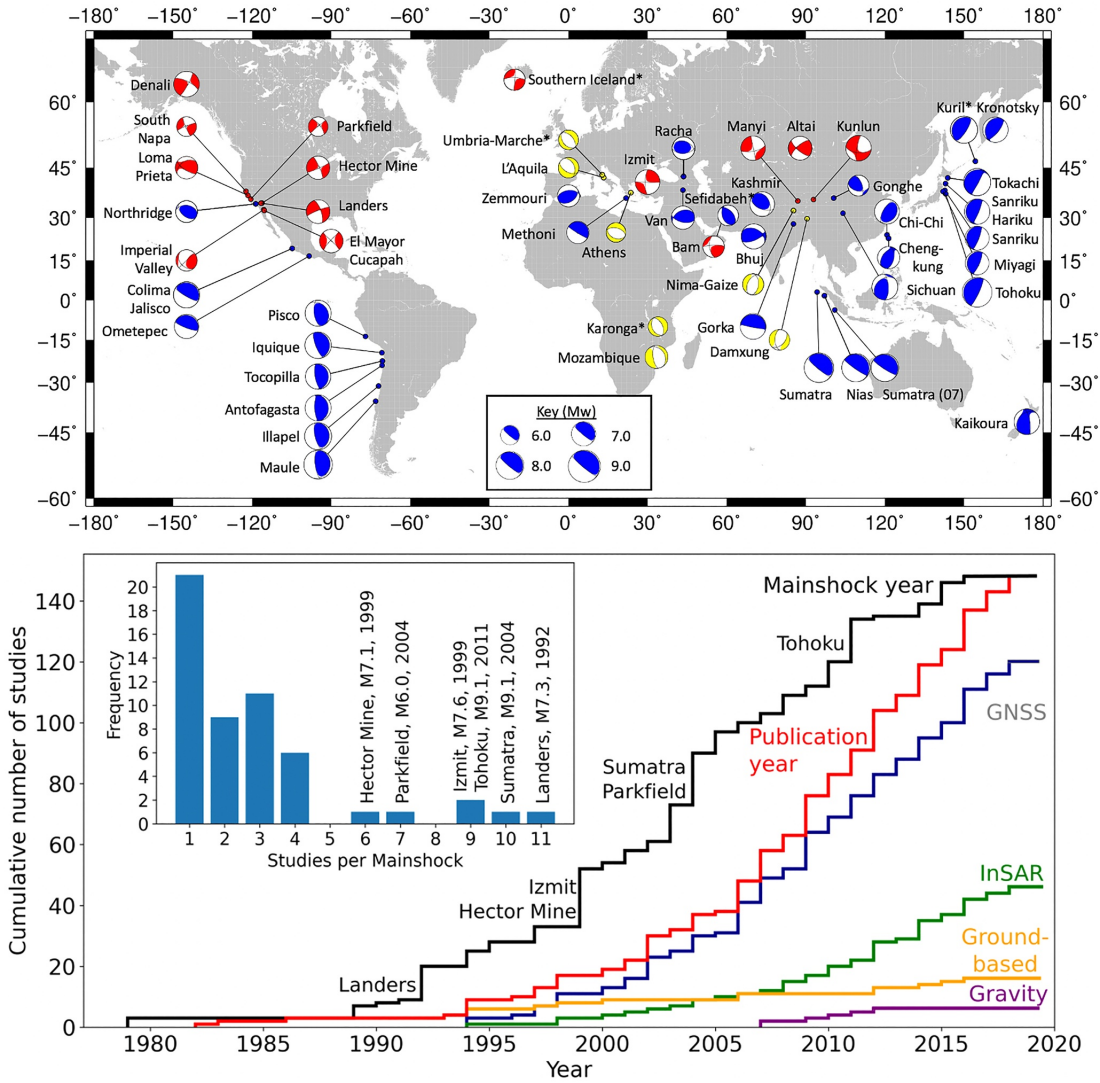
(a) The Global Centroid Moment Tensor focal mechanism solutions of the earthquakes in our database (red: strike slip, blue: thrust, yellow: normal, * indicates a mainshock sequence with the largest event shown). (b) The cumulative number of compiled studies is shown by year of mainshock and year of publication, and the cumulative use of data types by year of publication. (c) The frequency of studies per mainshock, with the most‐represented mainshocks, annotated, corresponding to large steps in panel (b).

The database contains 148 studies of afterslip following 53 mainshocks (doi:10.5281/zenodo.6414330). The earthquakes span *M*_*w*_ 6.0–9.1 and comprise 32 thrust, 14 strike‐slip, and 7 normal mechanisms (Figure 2a). Analysis of the GCMT catalog indicates that the database contains 100% of the *M*_*w*_9 earthquakes that occurred during the study period, 32% of *M*_*w*_8, 4% of *M*_*w*_7, and less than 1% of *M*_*w*_6 earthquakes. Smaller earthquakes are underrepresented in our compilation and those included may have a bias toward higher *M*_*rel*_ due to more readily detectable afterslip.

Studies vary in data practices and modeling methodologies. Overall, we categorize 18 geodetic analyses, 117 kinematic slip models, and 13 dynamic slip models. Approximately 41% of all studies considered only afterslip as a viable postseismic mechanism, 32% considered afterslip and viscoelastic relaxation, 3% considered afterslip and pore fluid factors, and 24% considered all three mechanisms. Figure 2b shows InSAR emerging and GNSS becoming dominant in the 1990s, with gravity‐based methods emerging more recently and ground‐based surveys scarcely used this century.

The database contains multiple afterslip studies for some earthquakes, although not every study proposes a moment estimate. There are multiple studies for 32 mainshocks, as shown in Figure 2c, and six particularly well‐studied examples: *M*_*w*_7.1 1999 Hector Mine (6 studies), *M*_*w*_6.0 2004 Parkfield (7), *M*_*w*_7.6 1999 Izmit (9), *M*_*w*_9.1 2011 Tohoku (9), *M*_*w*_9.1 2004 Sumatra (10) and *M*_*w*_7.3 1992 Landers (11). Overall, 95 studies provide a meaningful afterslip moment estimate as geodetic analyses generally cannot estimate moment and many kinematic and dynamic slip models do not explicitly calculate or give one. Eighty eight moment estimates come from kinematic slip models, whose methodologies are better constrained and thus more comparable.

**Figure 3:**
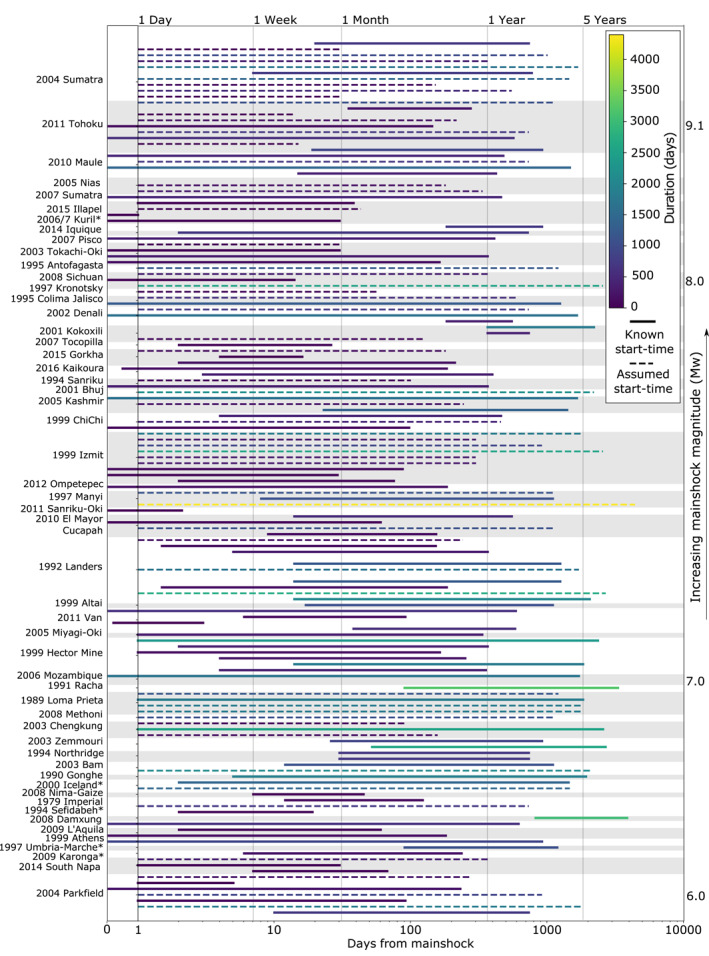
Temporal observation windows for all compiled studies, where available. The line length indicates the base‐10 logarithmic duration and the color gives linear duration. Dashed lines indicate studies without an explicitly provided start time, which we assume is 1‐day as most are continuous GPS.

The start times and durations of all studies are summarized in Figure 3, ordered by the mainshock. If afterslip velocities decay according to Equation 5 and this is linearly proportional to moment release rate, the cumulative moment release should be proportional to the logarithm of time, thus we present logarithmic time on the *x*‐axis. Most studies start within a few days of the mainshock, with approximately 1 day being the soonest and 2 years being the latest, and typically last for several months to around 2 years, with approximately 1 day being the shortest and 12 years being the longest. We explore the relationship between *M*_*rel*_ estimates and the start time and duration of observation in Sections 4.3 and 4.4 and discuss our findings in 5.3.

### 4.2 Afterslip Moment Scaling and Variation

**Figure 4:**
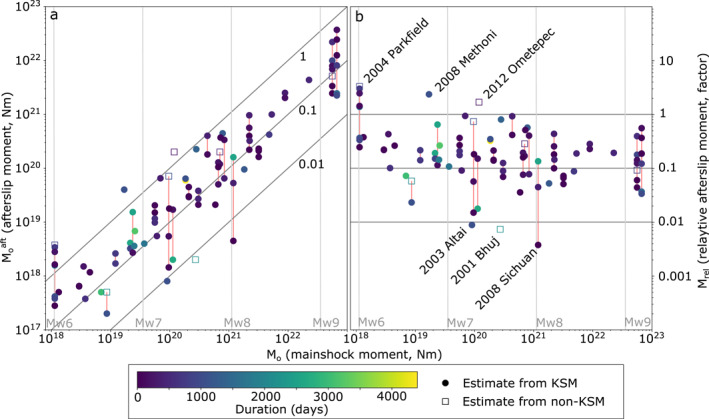
(a) Afterslip moment estimates against corresponding coseismic moments ($M_{o}^{aft}$ vs. *M*_*o*_) and (b) relative afterslip moment estimates against coseismic moment (*M*_*rel*_ vs. *M*_*o*_). The color scale shows the linear temporal duration of each model. Red bars link estimates for the same earthquake from different studies. The 88 circles denote the kinematic slip model estimates (KSMs) that are analyzed further. Relative afterslip moment estimates <1% and >100% are labeled.

Figure 4a gives afterslip moment estimates against the corresponding coseismic moment. For the 88 kinematic slip model estimates, the median Spearman's rank correlation coefficient between $M_{o}^{aft}$ and *M*_*o*_ is 0.91 after bootstrapping, with the 95% range between 0.89 and 0.93 (Figure 4a). This supports the hypothesis that aseismic afterslip moment scales with coseismic moment. We also note that the median Pearson's correlation coefficient between $\log\left(M_{o}^{aft}\right)$ and log(*M*_*o*_) is 0.92 after bootstrapping, with a gradient close to one. We infer near‐linear scaling of the afterslip moment with the coseismic moment for our mainshocks, which we discuss further in Section 5.1.

**Figure 5:**
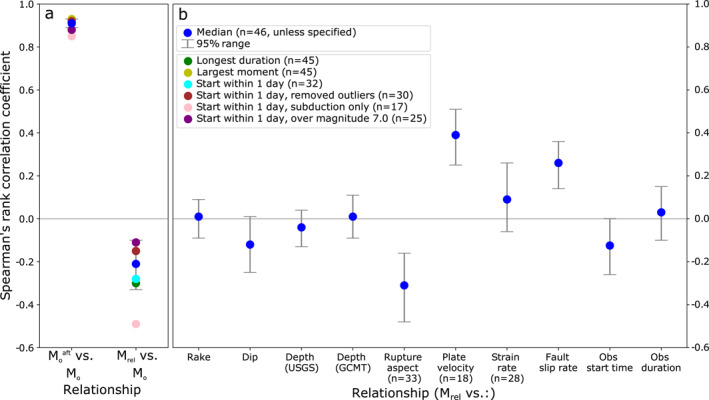
Median Spearman's rank correlation coefficients and 95% ranges for relationships tested. *n* = 46, with 88 data points bootstrapped over throughout, unless specified. (a) Absolute and relative afterslip moment against the coseismic moment. The correlation coefficients for reduced data sets are also shown, which do not require bootstrapping as there is only one data point per earthquake (n is given individually) and (b) relative afterslip moment against our tested metrics. The rake value is calculated slightly differently and is explained in Section 4.4.

The 95% range of Spearman's rank correlation coefficients better reflects variation due to bootstrapping than the variations in individual afterslip moment estimates. We analyze the uncertainty in some individual estimates in Section 4.3, but further test the robustness of the $M_{o}^{aft}$/*M*_*o*_ correlation by examining the reduced data sets defined in Section 3.4. These correlation coefficients range from 0.85 to 0.93, shown in Figure 5a, which is close to that obtained by bootstrapping over the entire data set, further supporting a robust and strong relationship.

Relative afterslip moment (*M*_*rel*_) varies over three orders of magnitude from <1% to >300% (Figure 4b). The median value for the 88 kinematic slip model estimates is 18% with an interquartile range of 9%–32%. Endmembers include two estimates below 1%: the *M*_*w*_7.2 2003 Altai earthquake (Barbot et al., 2008) and the *M*_*w*_8.0 2008 Sichuan earthquake (Shao et al., 2011), and five greater than 100%: the *M*_*w*_6.0 2004 Parkfield earthquake (Bruhat et al., 2011; Freed, 2007; Johanson et al., 2006; Langbein et al., 2006) and the *M*_*w*_6.8 2008 Methoni earthquake (Howell et al., 2017).

*M*_*rel*_ weakly and negatively correlates with the mainshock moment. The median Spearman's rank correlation coefficient for *M*_*rel*_ and *M*_*o*_ is −0.21 after bootstrapping, with the 95% range from −0.32 to −0.09. This could suggest that larger earthquakes are prone to less *M*_*rel*_, but this may be due to the publication bias, and is discussed further in Section 5.2. Correlation coefficients from the reduced data sets vary from −0.11 to −0.49 (see Figure 5a), likely because the data sets are smaller and thus less stable. The most outlying coefficient (−0.49) is from the smallest data set (*n* = 17), and removing a single outlying data point (for the *M*_*w*_6.8 2008 Methoni earthquake) highlights this instability as the correlation coefficient falls from −0.49 to −0.38. As the overall correlation between *M*_*rel*_ and *M*_*o*_ is much weaker than between $M_{o}^{aft}$ and *M*_*o*_, this motivates the investigation of other factors to account for variability in *M*_*rel*_.

### 4.3 Temporal Dependence and Uncertainty of Individual *M*_*rel*_ Estimates

**Figure 6:**
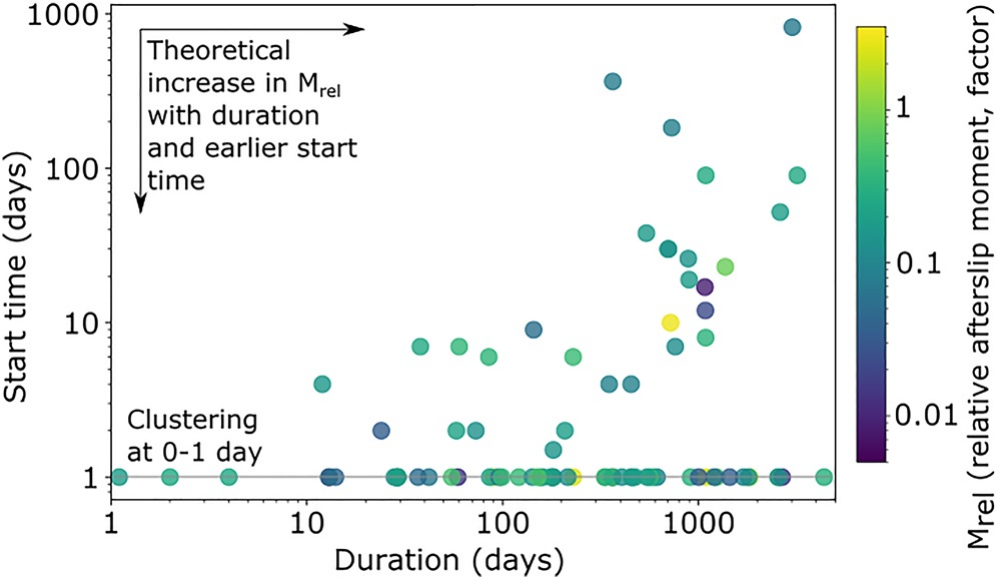
Observation start times and durations for all 88 kinematic afterslip models with relative afterslip moment estimates (*M*_*rel*_), are shown in color. Models with start times given before or on day one are shown at 1‐day, models without an explicitly provided start time begin on day 1.

Figure 6 shows the relationship between estimates of relative afterslip moment and the start time and duration of observation. The median Spearman's rank correlation coefficients after bootstrapping are −0.13 and 0.03, respectively, with 95% ranges of −0.24 to 0.00 and −0.09 to 0.16, respectively (Figure 5), indicating that across the data, there is no strong relationship between *M*_*rel*_ and observation start time and duration. This is surprising, given the theoretical temporal decay of afterslip: early and longer observation windows should result in greater afterslip moment estimates. We discuss the implications of this in Section 5.3.

**Figure 7:**
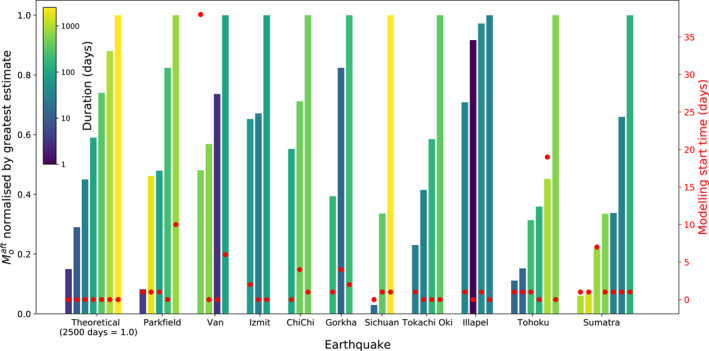
Afterslip moment estimates of the 10 mainshocks which have three or more kinematic slip model estimates. For each earthquake (shown across the *X*‐axis), different afterslip moment estimates are shown as bars, normalised to the largest and arranged from smallest to largest (scale is given on the left *Y*‐axis). The start time of the data used for each estimate is given by red circles (right *Y*‐axis) and the duration is given by color. A theoretical case of how the afterslip moment should grow with time (based on an assumed steady‐state, velocity‐strengthening decay behavior) is also shown for comparison. Here, estimates from different durations over which data were analyzed are shown in ascending order, reaching 1.0 at 2,500 days. Moment estimates of the Sichuan earthquake, for example, appear to follow the expected trend if afterslip estimates were solely determined by duration and onset of the analyzed dataset, whereas Parkfield estimates do not follow the expected trend, suggesting other modeling sources of uncertainty.

Figure 7 shows that different afterslip moment estimates following the same earthquake can vary considerably. If differences in observation start time and duration cannot explain these differences, this would imply significant modeling uncertainty. We analyze 10 earthquakes that have at least three afterslip moment estimates from different kinematic slip model studies. Estimates are normalized to the largest value for each earthquake to highlight the relative spread. We also present an expected, theoretical case in which afterslip is fully captured following an idealized earthquake by studies of different durations. To calculate this, we assume that afterslip velocity (Equation 5) linearly relates to the afterslip moment release rate, thus the integral with respect to time gives moment release. We assume the initial rate and constant *c* both equal one for simplicity and normalize to one unit of afterslip at 2,500 days to attain this idealized case.

For four earthquakes, the relative spread in individual afterslip moment estimates can be explained by differences in the temporal observation window. Afterslip moment estimates for the *M*_*w*_7.6 1999 Izmit and the *M*_*w*_7.6 1999 Chi‐Chi earthquakes are relatively well‐constrained within a factor of 2 (Figure 7), with larger estimates corresponding to increased observation duration or decreased observation start time. More varied afterslip moment estimates follow the *M*_*w*_8.2 2003 Tokachi Oki earthquake (varying by up to a factor of approximately 5) but correspond with the duration of observation. Similarly, following the *M*_*w*_8.0 2008 Sichuan earthquake, the smallest afterslip moment estimate is only 3% of the largest but corresponds to an observation duration of approximately two weeks compared to seven years. Both the theoretical and Sichuan case are normalized to one unit of afterslip at approximately 2,500 days, thus differences could be interpreted as (a) afterslip decaying faster after the Sichuan earthquake than the theoretical case, (b) the largest Sichuan estimate is erroneously high, (c) the two smaller Sichuan estimates are erroneously low, or (d) a combination.

For six earthquakes, the relative spread in individual afterslip moment estimates cannot be easily explained by differences in the temporal observation window. In two cases: the *M*_*w*_8.0 2011 Van and the *M*_*w*_8.3 2015 Illapel earthquakes, afterslip moment estimates are relatively well‐constrained by a factor of approximately two, but there are four cases where the relative spread is considerably greater: the *M*_*w*_6.0 2004 Parkfield, the *M*_*w*_7.8 2015 Gorkha, the *M*_*w*_9.1 2011 Tohoku and *M*_*w*_9.1 2004 Sumatra earthquakes. For example, following the Sumatra earthquake, the two longest duration studies produced afterslip moment estimates of approximately only 6% of the largest and 10% of the second largest (both of which happened to be among the shortest duration studies). This indicates that an individual afterslip moment estimate may be more than an order of magnitude too small or too large (i.e., <10 to >1000%). The extreme variation following the Tohoku and Sumatra earthquakes is surprising as these are among the best‐studied earthquakes and postseismic periods, and also suggests that uncertainty does not decrease with the coseismic moment. As six out of the 10 examples analyzed show spread in afterslip moment estimates which cannot be easily attributed to differences in observation start time or duration, we conclude that there is significant uncertainty associated with the modeling process.

This analysis indicates that the relative uncertainty in afterslip moment estimates can obscure the dependence we expect to see from either the observation start time or duration. For this reason, we do not attempt to normalize afterslip moment estimates for observational time window and instead consider individual afterslip moment estimates as given, but recognize potential for substantial uncertainty. Using the 10 analyzed earthquakes, we can assess the uncertainty of a typical afterslip moment estimate. The average mean and average variance of these 10 groups of estimates (each relative to the largest) is 0.62 and 0.1, respectively. Assuming, therefore, that a given afterslip moment estimate is 0.62 ± 0.1 of a full population of estimates, and that the best estimate solution lies somewhere in that population, the given estimate is likely within a factor of ∼two or three of the best estimate solution. However, in the most extreme case (as illustrated by the *M*_*w*_9.1 2004 Sumatra earthquake) estimates could be out by an order of magnitude. The sources and implications of this uncertainty are discussed in Section 5.3.

### 4.4 Factors Contributing to *M*_*rel*_ Variation

We investigate potential controls on relative afterslip moment by testing the hypotheses formed in Sections 2.2 and 2.3. Figure 5b summarizes the median Spearman's correlation coefficients between *M*_*rel*_ and our testable metrics after bootstrapping. These coefficients range from near zero to |0.39|, a weak to moderate correlation. The 95% ranges vary in width and reflect the full distribution of correlation coefficients from bootstrapping to indicate a sense of the robustness of the relationship.

**Figure 8:**
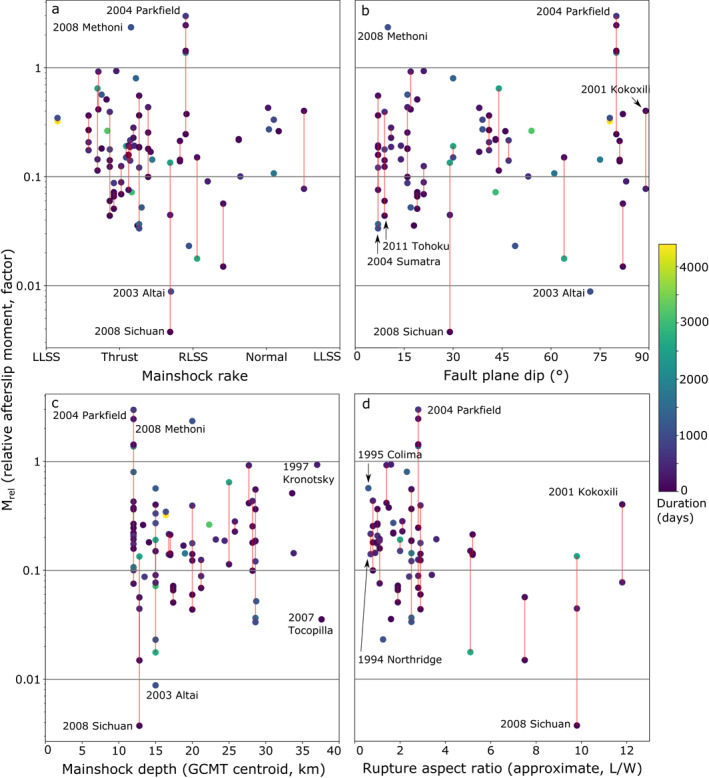
Relative afterslip moment (*M*_*rel*_) against (a) mainshock fault plane rake (we test the vertical component of rake, median Spearman's rank correlation coefficient: 0.01), (b) mainshock fault dip (−0.12), (c) mainshock centroid depth (0.04), and (d) approximate rupture aspect ratio (−0.31). a and b show USGS preferred solution moment tensor values, c shows Global Centroid Moment Tensor centroid depth values, and d uses models from the Earthquake Source Model Database (SRCMOD, for the 33 available events only). Red lines connect different estimates from the same earthquake and color indicates the temporal duration of each study.

Figure 8 shows *M*_*rel*_ against mainshock rake, fault dip, depth, and rupture aspect ratio. The correlation coefficients between *M*_*rel*_ and the vertical component of rake and dip are 0.01 and −0.12 (Figures 8a and 8b), respectively, with both 95% ranges crossing the zero coefficient baseline, indicating no obvious control on afterslip (GCMT and USGS rake and dip values were very similar). Whilst we show the actual rake value in Figure 8a, we test adjusted (semi‐circular not circular) values whereby thrust and normal mechanisms are endmembers and strike slip sits in between (i.e., right and left lateral slip are treated the same in the context of our hypothesis).

*M*_*rel*_ correlates with rupture aspect ratio but not with mainshock depth (Figures 8c and 8d). The median Spearman's rank correlation coefficients are −0.04 and 0.01 for the USGS and GCMT depths, respectively, indicating no obvious control on *M*_*rel*_. Figure 8d shows the approximate length‐to‐width rupture aspect ratio against *M*_*rel*_ for the 33 earthquakes for which a coseismic slip model was available. The associated median bootstrapped Spearman's rank correlation coefficient is a moderate −0.31 and has a 95% range entirely negative. As continental and subduction earthquake populations might behave differently in terms of aspect ratio (e.g., Ampuero & Mao, 2017), we also calculate the correlation coefficients for continental (−0.34) and subduction (−0.24) populations individually, but these are quite similar to one another and the overall average.

**Figure 9:**
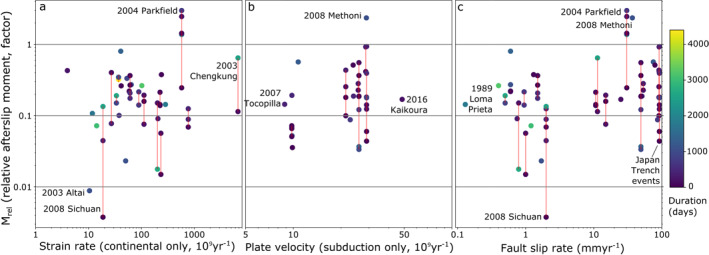
Relative afterslip moment (*M*_*rel*_) against (a) local strain rate for the 28 continental‐setting events (median Spearman's rank correlation coefficient: 0.09), (b) plate velocity for the 18 events broadly on a subduction interface (0.39) and (c) local fault slip rate for all 46 events (0.26). Red lines connect different estimates from the same earthquake.

Figure 9 shows *M*_*rel*_ against the local strain rate, plate velocity, and fault slip rate. These have correlation coefficients of 0.09, 0.39, and 0.26, respectively. The 95% ranges for the more strongly correlated plate velocity and fault slip rate relationships are also entirely above zero. The moderate relationship with plate velocity is for only 18 events on subduction interfaces, thus having less scope for interpretation as the fewer data points mean a less robust coefficient. However, the moderate relationship with fault slip rate is over the entire kinematic slip model data set of 46 earthquakes and 88 estimates, implying some robustness.

### 4.5 Afterslip Depth Analysis

**Figure 10:**
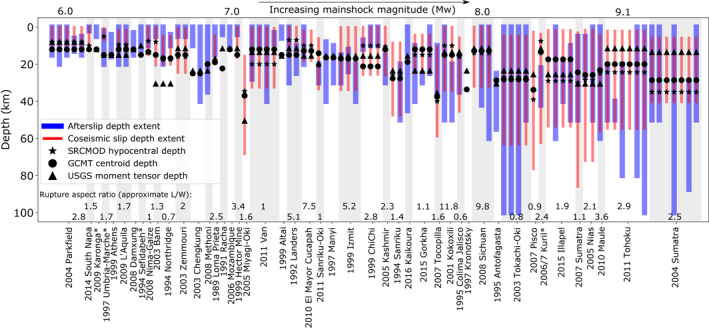
The approximate depth extents of aseismic afterslip for the 88 kinematic slip models studies and corresponding coseismic ruptures from SRCMOD coseismic slip models. Moment tensor depths from the USGS preferred solution, centroid depths from the Global Centroid Moment Tensor catalog, and rupture aspect ratios and hypocentral depths from SRCMOD coseismic slip models are also shown but may be erroneous in some cases (e.g., default values, not relocated). Not all afterslip depth extents, coseismic depth extents, hypocentral depths and rupture aspect ratios were available.

We investigate whether the occurrence of up‐ or downdip afterslip may be influenced by vertical rupture directivity, using the simple, one‐dimensional proxy of whether an earthquake's centroid is above or below the hypocenter. We conduct this analysis for 31 earthquakes for which we have: approximate afterslip and coseismic depth extents, hypocenter depths, and centroid depths. Figure 10 shows that in at least one study for each earthquake, afterslip and coseismic slip depths overlap by at least one km. We cannot comment on whether specific slip patches overlap, as this could be due to afterslip and coseismic slip distributions varying laterally. However, this at least indicates that rheological heterogeneity (i.e., deviations from simple, one‐dimensional slip stability models with constant depths, Figure 1) may be quite common in fault zones, especially as there is evidence of afterslip occurring throughout the entire coseismic slip depth range for approximately a third of the earthquakes in this analysis.

The relative depths of the centroid and hypocenter do not appear to influence the depth extent of afterslip. Twelve earthquakes have a hypocenter at least one km deeper than centroid, which we describe as net‐updip propagating. Six of these earthquakes show some evidence of afterslip significantly above coseismic rupture depths, whilst six do not. Additionally, five earthquakes that cannot be described as net‐updip propagating also show evidence of significant updip afterslip. At a threshold of five km, only five earthquakes qualify as net‐updip propagating and only two of these show evidence of significant afterslip updip of coseismic rupture.

Fourteen earthquakes have a hypocenter at least 1 km shallower than the centroid (net‐downdip propagating). Nine of these show some evidence of significant afterslip below coseismic rupture depths, but five do not. Similarly, seven earthquakes that cannot be described as net‐downdip propagating also show evidence of significant downdip afterslip. Again, at a threshold of 5 km, only five earthquakes can be described as net‐downdip propagating and only two of these show evidence of significant afterslip downdip of coseismic rupture. We, therefore, find no evidence that rupture directivity effects afterslip depth distribution, but our analysis is only one‐dimensional; effective slip analysis would be useful for more insight here, but digitized afterslip models are scarcely provided.

## 5 Discussion

### 5.1 Afterslip and Coseismic Moment Scaling

We find that afterslip and coseismic moment scale approximately linearly, with a gradient close to one. We explore what this finding might mean for the average slip and area of afterslip, through assumptions grounded in elastic theory and self‐similar rupture scaling. Whilst *M*_*rel*_ is distributed with an interquartile range of 0.09–0.32, to form a simple argument, we assume that *M*_*rel*_ can be approximated by a constant and rewrite Equation 1 as:

(7) $$M_{o}^{aft}=M_{rel}M_{o}.$$

We can substitute Equation 4 into 7 and assume that the shear modulus (*G*) remains approximately constant across the seismogenic and afterslip zones when compared to variations in *A* and $\bar{D}$. The average slip $\left({\bar{D}}^{aft}\right)$ and area (*A* ^*aft*^) of afterslip thus scale as:

(8) $$A^{aft}{\bar{D}}^{aft}∼A\bar{D}∼M_{o}.$$

We can consider the scaling of *A* ^*aft*^ and ${\bar{D}}^{aft}$ separately, by first considering an ‘activated area’ around a rupture that is primed for afterslip. For a simple circular rupture, stress change decays as the inverse of the distance cubed from the dislocation (Segall, 2010). Assuming that afterslip is entirely driven by coseismic static stress change, the distance (*d* ^*aft*^) to the minimum activating shear stress bounding the activated area (*A* ^*aft*^) scales with *M*_*o*_ as:

(9) $$d^{aft}∼M_{o}^{1/3},$$

(Marsan, 2005). Squaring the equation gives an area *A* ^*aft*^ that scales as:

(10) $$A^{aft}∼M_{o}^{2/3},$$

with proof given in the supplementary materials. Given Equation 8, ${\bar{D}}^{aft}$ must therefore scale as:

(11) $${\bar{D}}^{aft}∼M_{o}^{1/3}.$$

Our empirical finding (Equation 7) also allows us to substitute $M_{o}^{aft}$ into Equations 10 and 11 in the place of *M*_*o*_ (although the constants of proportionality change):

(12) $$A^{aft}∼{M_{o}^{aft}}^{2/3},$$

(13) $${\bar{D}}^{aft}∼{M_{o}^{aft}}^{1/3}.$$

Equations 12 and 13 refer to afterslip area, slip, and moment, but are essentially equivalent to well established coseismic scaling relations (e.g., Allen & Hayes, 2017; Blaser et al., 2010; Hanks & Bakun, 2002; Leonard, 2010, 2014; Murotani et al., 2013; Skarlatoudis et al., 2016; Somerville et al., 1999; Strasser et al., 2010; Wells & Coppersmith, 1994). Interestingly, Michel et al. (2019) also proposed that the area of slow slip events follow a relationship equivalent to Equation 12, implying that the area of afterslip and generic slow slip events may scale similarly.

We propose that the afterslip area and average slip approximately obey the scaling relations given by Equations 10 and 11. Afterslip moment grows by a combination of coseismic area and average slip, thus with the overall coseismic moment. We believe these scaling relations provide a good first order approximation of afterslip behavior, around which secondary factors can cause variation. Our results indicate that characteristics such as rupture aspect ratio and fault slip rate (a potential proxy for fault zone maturity and composition) may influence *M*_*rel*_ and therefore cause systematic deviations from this scaling.

### 5.2 Mainshock and Fault Setting Factors

In this section, we discuss the potential influences of mainshock characteristics, fault setting, and the broader earthquake cycle on *M*_*rel*_. We expect that afterslip occurrence is driven principally by how strongly and how much of the fault zone interface is velocity‐strengthening (*a* − *b* < 0), and that *a* − *b* is largely controlled by depth.

We find no evidence to support strong relationships between *M*_*rel*_ and mainshock rake, dip, or depth. This indicates that globally, neither mechanism nor depth overwhelmingly affects the afterslip moment and that our original hypothesis (that shallow ruptures and low fault dip angles may allow more afterslip) is not supported. However, our metrics may be overly simplistic or too insensitive to serve as good proxies for mainshock geometry, as they do not consider rupture shape, fault roughness, or kinks. It is also possible that any relationship is obscured by data or modeling uncertainties, which we discuss in Section 5.3.

The rupture aspect ratio may be a second‐order control on afterslip. The correlation coefficient between *M*_*rel*_ and rupture aspect ratio is moderate −0.31, with an entirely negative 95% range. Rupture aspect ratio depends on characteristics such as nucleation area, local seismogenic thickness, and whether an earthquake is sufficiently large to interact with the edges of the seismogenic zone (Ampuero & Mao, 2017; Weng & Yang, 2017), thus is inherently linked to coseismic moment. This is seen in Figure 10, in which the largest rupture aspect ratios belong to larger continental earthquakes, which generally saturate the seismogenic zone around *M*_*w*_6‐7 (Hawthorne et al., 2016) and then elongate with increasing magnitude. Subduction interface events generally occur on much wider and lower dip angle faults (Anderson, 1905), thus form a separate population of rupture aspect ratios in Figure 10, but the overall relationship is still seen. The correlation coefficients between *M*_*rel*_ and rupture aspect ratio for continental‐ and subduction‐only event populations are −0.34 and −0.24, respectively, similar to the overall value.

A relationship between rupture aspect ratio and *M*_*rel*_ may have more than one explanation. Hawthorne et al. (2016) suggested that larger, elongated ruptures may have a reduced capacity for relative afterslip compared to smaller, less elongated earthquakes, because of the relative size of the region surrounding the coseismic rupture that can undergo afterslip. Smaller and less elongate earthquakes may also generate more of their afterslip closer to the rheologically controlled seismic‐aseismic transition, which has greater scope to vary from location to location, than larger, more elongate ruptures which generate more of their afterslip closer to the temperature‐controlled brittle‐ductile transition. However, this argument assumes that the seismic‐aseismic transition is consistently above the brittle‐ductile transition, which may not hold everywhere. A greater scope for relative afterslip variability in smaller earthquakes, combined with a publication bias whereby smaller earthquakes with larger *M*_*rel*_ are preferentially studied, provides one explanation for the relationship we observe but implies that it is (at least in part) due to the publication bias. The dependence of shear stress change on rupture stress drop (Segall, 2010) may provide an alternative, physical argument. For the same coseismic moment, a larger area and presumably more elongated rupture will have a lower stress drop, and thus a smaller average stress concentration at its edges than a less elongated, more compact earthquake. Assuming that afterslip occurs generally downdip, this could imply that less elongate ruptures are able to generate more (downdip) afterslip than more elongate ruptures. Whilst rupture aspect ratio is not independent of the coseismic moment, *M*_*rel*_ is more strongly correlated to rupture aspect ratio than it is to *M*_*o*_, suggesting that rupture aspect ratio may provide some independent control on afterslip, although the specific reasoning is unclear.

*M*_*rel*_ correlates moderately with plate velocity and fault slip rate. Plate velocity, local strain rate, and fault slip rate are measures of deformation rate that we treat as proxies for fault maturity and high proportions of frictionally stable fault zone materials such as gouges and smoothed asperities (Choy & Kirby, 2004; Ikari et al., 2011). The moderate correlation between *M*_*rel*_ and plate velocity (0.39) is based on only 18 subduction interface earthquakes and is thus not particularly robust. The correlation between *M*_*rel*_ and strain rate for the remaining 28 continental events is a weak 0.09. The most significant finding is the moderate correlation between *M*_*rel*_ and fault slip rate (0.26) over the entire data set. Some geological evidence supports fault slip rate as a proxy for fault maturity (e.g., Goldberg et al., 2020; Manighetti et al., 2007), whilst reported slip rates may inadvertently be a good proxy of fault maturity, as measurements at immature faults may be systematically underestimated because the strain is less localized (Dolan & Haravitch, 2014). Regardless, the reported fault slip rate may be a reasonable first order proxy for maturity, and a weak to moderate indicator of *M*_*rel*_.

Endmember case examples can link high fault slip rates, specific geological characteristics, and high *M*_*rel*_. The highest *M*_*rel*_ estimates belong to the *M*_*w*_6.0 2004 Parkfield and *M*_*w*_6.8 2008 Methoni earthquakes, which have high fault slip rates, and high strain rates and plate velocities, respectively (see Figure 9). Near Parkfield, the fast creeping section of the San Andreas fault (Jolivet et al., 2015) contains several meters of highly velocity‐strengthening material gouge and talc‐bearing serpentinites (Johnson et al., 2006; Moore & Rymer, 2007; Savage & Langbein, 2008) which may explain the high *M*_*rel*_ and, perhaps, the relatively shallow afterslip observed (Bruhat et al., 2011; Johanson et al., 2006). In addition to this, Johanson et al. (2006) also posited that two *M*_*w*_5 aftershocks may have served to unpin an additional, adjacent fault section and trigger enhanced afterslip which explains the high *M*_*rel*_. High slip and strain rates might not be sufficient for abundant afterslip, however. Whilst our lowest *M*_*rel*_ earthquakes have relatively low fault slip rates (and strain rates and plate velocities), the *M*_*w*_6.8 2003 Chengkung and the Japan Trench earthquakes have the highest strain and slip rates, respectively, but more moderate *M*_*rel*_. Better estimates of lithology, rheology, and structure that can be used to describe the *a* − *b* profile at a fault would be helpful to further assess this dependence.

So far, we have only considered contemporary factors, but *M*_*rel*_ might vary over multiple earthquake cycles at a given fault. Simulations of different ruptures on the same fault patch have shown penetration to variable depths (Jiang & Lapusta, 2016; Shaw & Wesnousky, 2008), which could theoretically affect the fault area left primed for afterslip in future earthquakes, assuming that frictional stability is principally controlled by depth. Postseismic behaviors have even been shown to vary at the same fault patch in some of these simulations (e.g., Barbot et al., 2012). Furthermore, as stress conditions evolve with tectonic loading, exactly when an earthquake occurs could affect its afterslip. For example, regions adjacent to an ‘early’ earthquake might require less afterslip to catch up with the surrounding interseismic creep, than for a ‘late’ earthquake. Studies of several quasi‐periodic earthquake cycles at Parkfield have suggested this, indicating that the 1966 earthquake possibly produced more afterslip than the 1934 earthquake, which was ‘early’ (Segall & Du, 1993; Segall & Harris, 1987). However, data for these earthquakes and afterslip events are quite poor and the entire concept of quasi‐periodic seismic cycles is debated (Kagan et al., 2012). Interseismic coupling may be an important factor in determining *M*_*rel*_. More velocity‐strengthening fault surfaces surrounding rupture are likely to allow both more interseismic creep and afterslip and be less conducive to larger seismic ruptures, thus interseismic coupling could potentially be an indicator of afterslip potential, but requires reliable estimates at every fault.

In summary, *M*_*rel*_ does not appear to be overwhelmingly affected by earthquake mechanism, fault dip, or depth, but may be favored by higher fault slip rates and lower rupture aspect ratios. The uncertainty in *M*_*rel*_ estimates for the same event discussed in Section 4.3 highlights that any of these relationships may be obscured by data and modeling uncertainty. We discuss this further below, but perhaps stronger or additional relationships could be established by observing more earthquakes in the same locations over time and attempting to model the afterslip in a systematic way.

### 5.3 Data and Modeling Factors

We have identified significant uncertainty in afterslip moment estimates that must be due to data and modeling factors. In this section, we explore factors within the modeling methodology that might have led to (a) the lack of strong relationships between *M*_*rel*_ and the start time and duration of observation across global and individual earthquake scales and (b) the observed variability in afterslip moment estimates. The identification of this uncertainty should lead to a more informed analysis of afterslip models and perhaps an effort to standardize afterslip modeling methodology to improve model comparability and help us to better understand aseismic afterslip.

Afterslip moment should tend toward an asymptotic limit with earlier and longer observation windows, but we did not see strong evidence for this globally. The theoretical importance of observational duration is highlighted in Figure 7 by the synthetic afterslip decay case and can also be seen within some individual examples (e.g., estimates for the *M*_*w*_8.0 2008 Sichuan earthquake). The importance of an early start time is highlighted clearly in studies such as Jiang et al. (2021), who proposed that *M*_*rel*_ may have reached 34% within 24 hr of the 2004 *M*_*w*_6.0 Parkfield earthquake. There are several potential explanations for the lack of these relationships in the data. First, other potential dependencies such as rupture aspect ratio and fault slip rate may contribute to obscuring temporal relationships in analysis across different regions. Secondly, as afterslip can decay at different rates across different regions (Ingleby & Wright, 2017), 3 months of observation following one earthquake might capture a greater fraction of its afterslip than in 1 year following another; thus global‐scale correlations between *M*_*rel*_ and observation duration may be obscured. However, if either (or both) of these arguments are true, we would still expect to see correlations within different estimates following the same earthquake and within similar regions. In Section 4.3, we show that this is often not the case and that modeling must therefore be a significant source of afterslip estimate uncertainty, ranging from a typical factor of ∼two or three to over an order of magnitude.

A number of modeling choices may contribute to more variable afterslip moment estimates. A total of 47 of the 88 kinematic afterslip models we analyze do not properly account for or reasonably consider additional postseismic mechanisms (i.e., they did not model viscoelastic relaxation or pore fluid effects, or indicate why this is not required), which could lead to erroneous afterslip moment estimates (e.g., McCormack et al., 2020; Sun & Wang, 2015). The implications of not considering viscoelastic relaxation could be especially significant. For example, following the *M*_*w*_8.0 2008 Sichuan earthquake, M. Wang et al. (2021) suggested that an afterslip‐only model produced an afterslip moment estimate several times that of a model that included viscoelasticity. Conversely, they also suggested that not considering afterslip in viscoelastic relaxation models can lead to incorrect inferred effective viscosities. Additional examples where the trade‐off of afterslip and viscoelasticity in models may be significant include following the *M*_*w*_7.8 2015 Gorkha earthquake (e.g., B. Zhao et al., 2017), the *M*_*w*_7.9 2001 Kokoxili earthquake (e.g., D. Zhao et al., 2021) and the great *M*_*w*_9.1 2011 Tohoku (e.g., Sun & Wang, 2015) and *M*_*w*_9.1 2004 Sumatra (e.g., F. Pollitz et al., 2008) subduction thrust earthquakes. This may explain why uncertainty does not decrease with coseismic moment. When considering both mechanisms, separating their respective contributions is also a difficult problem, particularly in the lower crust (Jacobs et al., 2002; Luo & Wang, 2021). Modeling additional mechanisms also requires more complex rheological model spaces, thus additional free parameters (e.g., Bruhat et al., 2011; Muto et al., 2016; B. Zhao et al., 2017). The validity of different rheological spaces is an ongoing debate and an obvious source of uncertainty. Bedford et al. (2016) argue that the homogeneous, elastic half‐space is established, acceptable and useful for modeling afterslip, whilst others (e.g., Hearn & Burgmann, 2005; Sun & Wang, 2015) propose that layered elastic and viscoelastic half‐spaces are more valid and can recover more afterslip. Finally, the failure to remove the deformation signal due to aftershocks could lead to overestimates of the afterslip moment and distorted spatial models (Lange et al., 2014). Aftershocks are commonly ignored in afterslip studies due to a comparatively small cumulative moment (e.g., Diao et al., 2018). However, if particularly large aftershocks are not explicitly accounted for, this could amount to significant errors in afterslip moment estimates: we adjusted one estimate by Gahalaut et al. (2008) by 47%, but only because they explicitly stated this. We encourage researchers to reserve the term afterslip for a specific phenomenon outlined in Section 2, rather than generic postseimsic deformation.

Uncertainty surrounding different methodological practices remains a significant barrier to comparing afterslip models. More general sources include the non‐uniqueness and regularization inherent to the inversion process (Menke, 2018; Scales & Tenorio, 2001), approximations of topography and fault geometry, and data sensitivities, resolution, and distribution (Marchandon et al., 2021). For example, InSAR may often miss early afterslip or struggle to detect far‐field deformation resulting from deep afterslip (Marchandon et al., 2021; Wimpenny et al., 2017). Many of the modeling choices outlined in this section are compiled and summarized in our database for further investigation. A push toward the standardization of kinematic afterslip modeling methods would help improve the comparability of afterslip models and allow better deductions of afterslip behaviors, fault zone structure, and the relationship between afterslip and aftershock sequences. Many specific best modeling practices are still unclear and require further research before implementation, such as how appropriate different rheological models spaces are for modeling postseismic mechanisms. However, we recommend transparency and explicit quantification of parameters and uncertainties, the provision of digital afterslip models (if possible) for further analyses, and a push toward standardized data quality and temporal observation windows (i.e., an effort to start observation periods as early as possible and ensure a long duration), while recognizing that this is not always possible.

## 6 Conclusion

We compile a database of 148 afterslip studies after 53 earthquakes, containing detailed information on mainshock characteristics, modeling methods, and outputs (doi:10.5281/zenodo.6414330). By analyzing a subset of 88 well‐constrained kinematic slip models, we find that: (a) coseismic moment is the principal control on the ensuing afterslip moment, which scales near‐linearly with a median value of 18% of the coseismic moment, (b) relative afterslip moment (*M*_*rel*_) varies from less than 1% to over 300% of the coseismic moment, with an interquartile range of 9%–32%, (c) global variation in *M*_*rel*_ cannot be accounted for by variation in factors such as fault dip, rake, and depth, (d) global variation in *M*_*rel*_ may be related to rupture aspect ratio and fault slip rate (which might be indicative of fault maturity), (e) there is an unexpected lack of strong, correlation between *M*_*rel*_ and the start time and duration of observation window on global scales, which could be obscured by other relationships or because afterslip decays sufficiently differently in different regions. However, as differences in start time and duration of observation window cannot always account for different *M*_*rel*_ estimates by different studies following the same earthquake, we infer that: (f) there is significant, up to order‐of‐magnitude uncertainty in afterslip moment estimates related to the modeling process, which currently provides a barrier to systematic comparison. Our database and analysis help expose the current uncertainty in afterslip moment estimates and hopefully encourage the community to consider standardizing processes to provide increased ability to compare studies. Such comparisons can better constrain variability in afterslip behaviors, and deduce their controls. Understanding the controls on afterslip moment may allow the eventual incorporation of afterslip as a source of postseismic stress transfer in aftershock sequence hazard models.

## Supporting information

Supporting Information S1

Data Set S1

## Data Availability

Data used in this study are accessible through U.S. Geological Survey (2017), Dziewonski et al. (1981); Ekström et al. (2012), Mai and Thingbaijam (2014), Styron and Pagani (2020), Kreemer et al. (2014), UNAVCO (2021), as indicated in text, and through the database (doi:10.5281/zenodo.6414330).
