# Supplementary material for: Afterslip Moment Scaling and Variability From a Global Compilation of Estimates
Source: J Geophys Res Solid Earth. 2022 Apr 12;127(4):e2021JB023897. doi: 10.1029/2021JB023897 (PMC9287082; doi:10.1029/2021JB023897)
Supplement: Supplementary file 1 — Supporting Information S1 [file JGRB-127-0-s001.pdf]

Supporting Information for "Afterslip Moment Scaling and Variability from a Global Compilation of Estimates"

R. M. Churchill[1 *], M. J. Werner[1], J. Biggs[1], Å. Fagereng[2]

[1]School of Earth Sciences, University of Bristol, Bristol, UK

[2]School of Earth and Environmental Sciences, Cardiff University, Cardiff, UK

Contents of this file

1. Text S1: Proof that $A^{aft} \propto M_o^{\frac{2}{3}}$

2. Figure S1: Accompanying Figure for Text S1

3. Text S2: An outline of the format of the database compiled

4. Table S1: Median Spearman's correlation coefficients for M_o^{aft} vs M_o and M_{rel} vs M_o

5. Table S2: Median Bootstrapped Spearman's correlation coefficients for M_{rel} versus potential controlling factors

Additional Supporting Information (Files uploaded separately)

1. Database "Churchill2022AfterslipDatabase.xlsx".

---

[*]robert.churchill@bristol.ac.uk

Introduction

This document includes Text S1, a basic mathematical proof supplementary to discussion within our paper. Also included is Figure S1, which accompanies Text S1 and shows this proof graphically. Next, Text S2 is an outline of the database format. The database "Churchill2022AfterslipDatabase.xlsx" is uploaded separately and also hosted online (see paper for details). Finally, Tables S1 and S2 summarise correlation coefficients from the study.

Text S1. Proof that $A^{aft} \propto M_o^{\frac{2}{3}}$

This is a basic mathematical proof that the area of a halo of afterslip A^{aft} is proportional to $M_o^{\frac{2}{3}}$, where M_o is coseismic moment for an idealised, simple circular rupture (see Figure S1). We first assume that:

$d^{aft} \propto M_o^{\frac{1}{3}}$ and $r \propto M_o^{\frac{1}{3}}$.

The area of the halo of afterslip (as shown in Figure S2) is given by:

$$A^{aft} = \pi(r + d^{aft})^2 - \pi r^2,$$

which can be simplified by substituting in the first two equations:

$$A^{aft} \propto \pi(M_o^{\frac{1}{3}} + M_o^{\frac{1}{3}})^2 - \pi(M_o^{\frac{1}{3}})^2,$$

thus:

A^{aft} is proportional to $\pi 4 M_o^{\frac{2}{3}} - \pi(M_o^{\frac{2}{3}})$,

and as the constants are not important in proportionality scaling:

$$A^{aft} \propto M_o^{\frac{2}{3}}$$

Text S2. An outline of the format of the database compiled

We present the database as the Microsoft Excel file "Churchill2022AfterslipDatabase.xlsx". Fields first give the mainshock ID, the mainshock date and time (UTC) and the afterslip study citation. Fields then summarise the USGS preferred W-phase moment tensor (M_{ww}) solution: mechanism, magnitude, driving moment, depth, parent catalog, latitude, longitude, dip and rake, and the GCMT mainshock solution: latitude, longitude, centroid depth, mainshock moment, strike, dip, rake, and information on a coseismic slip model: hypocentral depth, approximate upper and lower depth extent, approximate rupture width, length and aspect ratio, and a citation for the model. For the six mainshock sequences detailed in the paper, all fields reflect only the largest event, except for the summed driving moment field.

Fields then summarise the afterslip study: year, other postseismic mechanisms considered (viscoelastic relaxation, pore fluid effects), postseismic mechanisms deemed responsible, broad modelling type (geodetic analysis, kinematic slip model or dynamic slip model), data types used (ground-based, GNSS, InSAR, gravity), approximate top and bottom of model domain, rheological structure (elastic half-space, layered elastic half-space, viscoelastic half-space with an elastic lid, complex shear zone), shear modulii, observation end and start time, afterslip moment estimate (preferred model), upper and lower moment bound or error, M_{rel}, and approximate upper and lower depth extent of most afterslip. Field explanations are also provided as comments in the field headers.

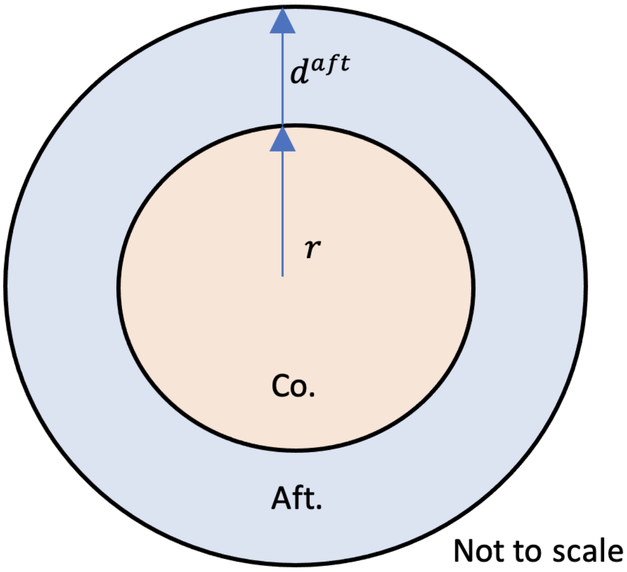

Figure S1. Accompanying Figure for Text S1. a simplified circular coseismic (co.) rupture of radius r, with a halo of afterslip (Aft.), d^{aft} wide.

Table S1. Median Spearman's correlation coefficients for M_o^{aft} vs M_o and M_{rel} vs M_o

Data set	Earthquakes (n)	M_o^{aft} vs M_o	M_{rel} vs M_o
Kinematic slip model estimates[a]	46	0.91 (0.89, 0.93)	-0.21 (-0.32, -0.21)
Longest duration study	45	0.91	-0.30
Largest M_o^{aft} estimate	45	0.93	-0.11
Longest studies starting on or before 1 day	32	0.92	-0.28
- Without endmembers	30	0.92	-0.15
- Subduction events only	17	0.85	-0.49
- $> M_w 7.0$ events only	25	0.88	-0.11

[a] Bootstrapping is used as there are 88 moment estimates for 46 earthquakes.

Table S2. Median Bootstrapped Spearman's correlation coefficients for M_{rel} versus potential contributing factors

Property	Median Spearman's rank correlation coefficient	95% range
Mainshock rake	0.01	-0.07, 0.11
Mainshock fault dip	-0.12	-0.25, 0.01
Mainshock depth (USGS)	-0.04	-0.12, 0.05
Mainshock depth (GCMT)	0.01	-0.09, 0.11
Mainshock rupture aspect (n=33)[a]	-0.31	-0.46, -0.14
Plate velocity (n=18)[a]	0.39	0.27, 0.53
Strain rate (n=28)[a]	0.09	-0.08, 0.24
Slip rate	0.26	0.16, 0.38
Observation start time (days)	-0.13	-0.24, 0.00
Observation duration (days)	0.03	-0.09, 0.16

[a] n denotes the number of earthquakes for these reduced data sets.
